# Toxic Methemoglobinemia Treated With Ascorbic Acid: Case Report

**DOI:** 10.5812/ircmj.12718

**Published:** 2013-12-05

**Authors:** Hatice Topal, Yasar Topal

**Affiliations:** 1 Department of Pediatrics, Mugla Sıtkı Kocman University, Faculty of Medicine, Mugla, Turkey

**Keywords:** Methemoglobinemia, Prilocaine, Ascorbic Acid

## Abstract

Methemoglobinemia is a disorder characterized by the presence of a higher than normal level of methemoglobin. Prilocaine which is one of the oxidizing local anaesthetics is widely used in many local procedures. The first choice of treatment of complications due to the use of these local anaesthetics is methylene blue, while ascorbic acid is the alternative choice. The side effects of metilen blue restrict its usage in some special conditions. Ascorbic acid is a good alternative drug with limited experience in methemoglobinemia. We present a case of a methemoglobinemia treated with ascorbic acid successfully to emphasize the use of ascorbic acid as an alternative method.

## 1. Introduction

Methemoglobinemia is a disorder characterized by the presence of a higher than normal level of methemoglobin (metHb, i.e. ferric [Fe3+] rather than ferrous [Fe2+] hemoglobin) in the blood. It is caused as a result of administrating oxidizing agents with the associated oxygen–hemoglobin dissociation curve therefore shifted to the left. Methemoglobinemia can be congenital, but its acquired form is more often (1). The acquired form might be seen after consumption of some foods and additives, and exposure to certain chemicals and drugs. Although local anaesthetics which are widely used in hospitalized and outpatients are usually safe for use, sometimes can cause life threatening methemoglobinemia (2). Prilocaine is one of the most frequently used anaesthetics in children, rarely causing methemoglobinemia (3, 4). Here, we present a case of a methemoglobinemia treated with ascorbic acid successfully to emphasize the use of ascorbic acid as an alternative method especially in regions where glucose-6-phosphate dehydrogenase (G6PD) deficiency is more frequent.

## 2. Case Presentation

A previously healthy 70-day-old male was brought to our clinic (Mugla Sitki Kocman University, Medicine Faculty, Department of Pediatrics, Mugla/Turkey; February 2012) with cyanosis on the perioral region, trunk and hands, three hours after he was administered locally prilocaine (Cytanest 2%; Astra Zeneca, Istanbul, Turkey) for circumcision. His previous health history and family history were unremarkable. On physical examination, temperature of 36 °C, blood pressure of 90/70 mm Hg, heart rate of 128 beats per minute regular with normal pulses and respiratory rate of 44 beats per minute with normal breathing. Oxygen saturation level was 76% (with pulse oximeter) on room air while he had prominent cyanosis on perioral region and the extremities. The rest of the physical examination was normal.

Intravenous line was obtained, and the blood collected for laboratory analysis had chocolate-color appearance. The laboratory results analysed by routinely calibrated equipments revealed; Hb 11 g/dl, WBC 9310/μL, PLT 622000/μL and all other biochemistry values were in normal range (Symex XT 2000i,Roche Diagnostics, Mannheim, Germany). Arterial blood gases (GEM premier 3000,instrument laboratory, Italy, calibrated in january 2012) revealed pH 7.43, pCO2 41 mmHg, pO2 97 mmHg and bicarbonates 28 mEq/L, and methemoglobin 24.5% (Table 1). After obtaining blood samples to check the methemoglobin levels, 300 mg IV ascorbic acid was slowly administered in 24 h. After 10 min, oxygen saturation began to increase and an hour later reached its normal levels. Methemoglobin levels were found to be 24.5% before ascorbic acid administration. After 24 hour of administration, methemoglobin level was seen to be 2%.

**Table 1.** Laboratory and Clinical Variables.

Laboratory	Results
**Hemoglobin**	11 gr / dL
**Leucocyte**	9310 / μL
**Platelet**	622000 / μL
**PCO_2_**	41 mmHg
**PO_2_**	97 mmHg
**HCO3**	28 mEq / L
**PH**	7.43
**SPO_2_**	76 %
**Methemoglobin**	24.5 %
**Clinical variables**	
**Breath sounds**	44 / min
**Heart beats**	128 / min
**Body temperature**	36 °C
**SPO_2_**	76%

## 3. Discussion

Methemoglobinemia was considered when patient’s saturation was below 80% and not-increased after 100% of O_2_ administration; the chocolate-color appearance of the blood and high oxygen pressure in arterial blood gases. Normally, methemoglobin levels are < 1% of total hemoglobin and has no oxygen carrying capacity but high oxygen affinity. Therefore tissue oxygenation is decreased, and cyanosis develops and the oxygen dissociation curve shifts to the left (5). The amount of methemoglobin in erythrocytes is defined by the balance of the increasing effects of oxidizing agents and the decreasing effects of the reductive systems in erythrocytes. Methylene blue increases NADH methemoglobin reductase enzyme activity (6). Acquired methemoglebinemia develops as a result of many drugs with oxidizing effects, and when methemoglobin levels are above the reductive capacity of the erythrocytes (7, 8). Most important drugs with oxidizing features are nitroglycerine, dapsone, sulfonamides, primacine, phenytoin, phenacetin, and local anaesthetics. It is known that primarily bezocain and many local anaesthetics cause methemoglobinemia. In local procedures such as circumcision the risk of methemoglobinemia due to prilocaine increases when younger the patient and higher the dose administered, as it was in our case (9, 10). In young children, particularly in those younger than 6 months, the underdeveloped enzyme system is one of the factors responsible for the increase in methemoglobin levels. In peripheral local anaesthetic procedures, higher dose of prilocaine, low volume but high concentration and small age increase the risk of methemoglobinemia related to the use of prilocaine (11). It is advised to avoid the use of prilocaine in children other than cutaneous applications. Bupivacaine might be a better alternative method (3). More than 90% of the patients do not show signs of methemoglobinemia due to local anaesthetics. Generally 10 - 20% of methemoglobin levels are well tolerated while symptoms are detected on higher rates. Methemoglobin levels higher than 70% lead to coma and death (12). The prognosis of patients surviving the 2nd day is generally well. On admittance our patient did not have any cardiac or respiratory problem other than cyanosis.

Absence of respiratory and cardiac problems, cyanosis and oxygen saturation not improved despite oxygen administration led us think the diagnosis of methemoglobinemia in our case. High partial oxygen pressure despite low saturation is due to the diluted oxygen in the blood not reflecting the hemoglobin saturation (13).

Prilocaine causes methemoglobinemia through its metabolite o-toluidine. The increase in this metabolite is responsible for hemoglobin oxidation. Prilocaine, used in therapeutic doses (1-2 mg/kg) is known to increase the level of methemoglobin. Methemoglobinemia level might increase to life threatening levels in newborns or infants who generally have underdeveloped enzymes (14, 15). The symptomatic methemoglebinemia in our case can be explained with its dose of 30 mg (5 mg/kg) which is toxic and additionally the age of the patient being 70 days.

Methylene blue is the treatment of choice for patients other than those with G6PD deficiency. Ascorbic acid should be used in those with G6PD deficiency because even therapeutic doses of methylene blue can lead to hemolysis of lead to he­molysis of erythrocytes (16). We used ascorbic acid because we were not able to obtain methylene blue and the oxygen saturation levels reached normal range after one hour of ascorbic acid administration. In cases of renal impairment or G6PD deficiency, the use of ascorbic acid should be considered as an alternative. Even though the rate of G6PD deficiency is not precisely known in our region, it is an often encountered problem. Therefore, the use of methylene blue might be risky and the use of ascorbic acid could be appropriate. Although hemolysis can be seen particularly in premature babies we did not encounter such a problem in our patient. Although, there is well known experience with methylene blue, ascorbic acid might be used as an alternative treatment as it has no major side effects and can be easily found.
